# The beneficial effects of nutraceuticals and natural products on small dense LDL levels, LDL particle number and LDL particle size: a clinical review

**DOI:** 10.1186/s12944-020-01250-6

**Published:** 2020-04-11

**Authors:** Sepide Talebi, Mohammad Bagherniya, Stephen L. Atkin, Gholamreza Askari, Hossein M. Orafai, Amirhossein Sahebkar

**Affiliations:** 1 Students’ Research Committee, Isfahan University of Medical Sciences, Isfahan, Iran; 2 Department of Community Nutrition, School of Nutrition and Food Science, Food Security Research Center, Isfahan University of Medical Sciences, Isfahan, Iran; 3 Weill Cornell Medicine Qatar, Doha, Qatar; 4 Food Security Research Center and Department of Community Nutrition, School of Nutrition and Food Science, Isfahan University of Medical Sciences, Isfahan, Iran; 5 Department of Pharmaceutics, Faculty of Pharmacy, Al-Zahraa University, Karbala, Iraq; 6 Halal Research Center of IRI, FDA, Tehran, Iran; 7 Biotechnology Research Center, Pharmaceutical Technology Institute, Mashhad University of Medical Sciences, Mashhad, Iran; 8 Neurogenic Inflammation Research Center, Mashhad University of Medical Sciences, Mashhad, Iran

**Keywords:** Phytochemical, Medicinal plant, Nutrition, Lipoprotein, Atherosclerosis

## Abstract

Cardiovascular diseases (CVDs) are globally the major causes of morbidity and mortality. Evidence shows that smaller and denser low-dense lipoprotein (sdLDL) particles are independent atherogenic risk factors for CVD due to their greater susceptibility to oxidation, and permeability in the endothelium of arterial walls. sdLDL levels are an independent risk factor and of more predictive value than total LDL-C for the assessment of coronary artery disease and metabolic syndrome. Functional food ingredients have attracted significant attention for the management of dyslipidemia and subsequently increase cardio-metabolic health. However, to date there is no study that has investigated the effect of these bioactive natural compounds on sdLDL levels. Therefore, the aim of the present review is to summarize the evidence accrued on the effect of special dietary ingredients such as omega-3 polyunsaturated fatty acids, nutraceuticals and herbal medicines on the levels of sdLDL, LDL particle number, and LDL particle size. Based on the results of the existing clinical trials this review suggests that natural products such as medicinal plants, nutraceuticals and omega-3 fatty acids can be used as adjunct or complementary therapeutic agents to reduce sdLDL levels, LDL particle numbers or increase LDL particle size and subsequently may prevent and treat CVD, with the advantage that theses natural agents are generally safe, accessible, and inexpensive.

## Introduction

Cardiovascular diseases (CVDs) are a global problem and the leading cause of morbidity and mortality [1]. It is projected that the prevalence of death from CVDs will be greater than 23.6 million people by 2030 [2]. Multiple risk factors, including unhealthy diet, physical inactivity, diabetes, dyslipidemias, and hypertension are considered to be modifiable factors in CVD [3]. It is well known that progression of CVD and increased atherosclerotic risk are positively linked with dyslipidemia. Hence, optimal levels of lipid profile play an important role in reducing atherosclerotic process [4].

**Fig. 1:**
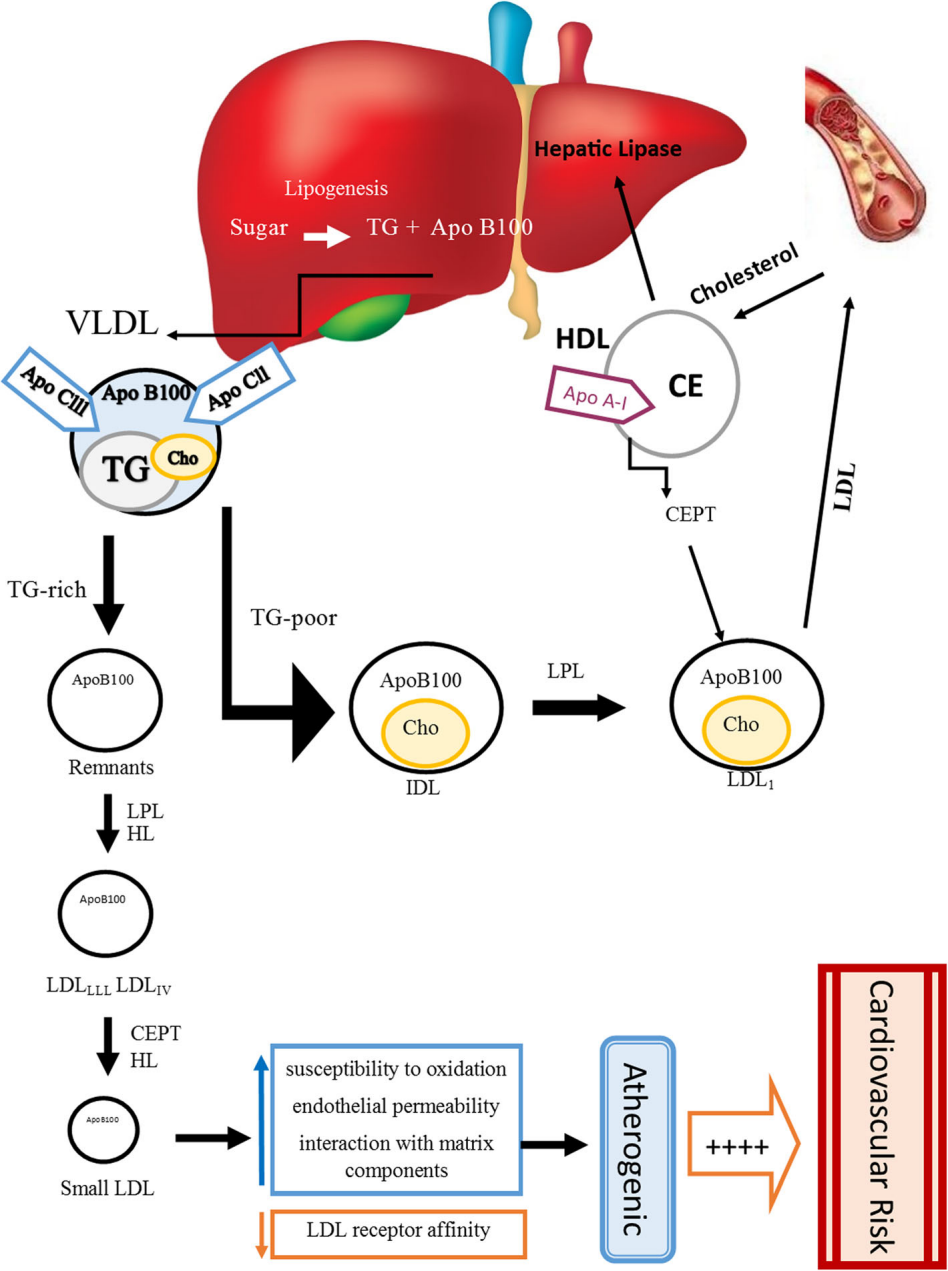
Schematic summary of pathways of endogenous lipid metabolism and pathways of the atherogenic and anti-atherogenic lipoproteins. sdLDL: small dense low density lipoprotein, Apo: apolipoprotein, VLDL: very-low-density lipoprotein, IDL: intermediate-density lipoprotein, LDL: low-density lipoprotein; LDL-R: low-density lipoprotein receptor, HDL: high-density lipoprotein, TG: triglycerides, CE: cholesteryl esters, +: increased risk

LDL-C is one type of lipoprotein that composed of a heterogeneous group of molecules with different sizes, density, lipid composition and physical properties. Smaller and denser LDL particles are considered as an atherogenic risk factor for CVD due to their greater susceptibility to oxidation, and their permeability through the endothelium of arterial walls [5, 6]. Large buoyant LDL particles (diameter ≥ 25 nm) have been considered as phenotype pattern A and small, dense LDL particles with sizes 19.0–20.5 nm have been considered as phenotype pattern B [7–9]. The positive link between individuals with high triglycerides and low HDL-C concentrations and LDL pattern B has been established, which are the features of the metabolic syndrome [10]. Small dense low-density lipoprotein (sdLDL) particles are a subfraction of LDL that are characterized by changes in their chemical content, and they are free of cholesterol and cholesterol ester, as well as having a decrease in phospholipid content, whilst the triglyceride content remains unchanged [11]. sdLDL is produced by very low-density lipoproteins (VLDL) in triglyceride-rich conditions derived from the liver. VLDL particles are initially converted to LDL class III and IV by the lipoprotein lipase (LPL) enzyme. Subsequently, the enzyme of cholesteryl ester transfers protein (CETP) with the help of hepatic lipase (HL) transfer TG to sdLDL particles leading to increased levels of sdLDL particles. At low TG levels, VLDL particles are converted to intermediate-density lipoprotein (IDL) and large LDL subclasses (Fig. 1) [5, 12]. Previous studies have shown that sdLDL levels are an independent risk factor and more predictive than total LDL-C for the assessment of coronary artery disease [13, 14] and metabolic syndrome [15]. sdLDL particles play a crucial role in the etiology of ischemic heart disease (IHD) and coronary heart disease (CHD) [16, 17]. Guidelines of the Association of Clinical Endocrinologists (AACE) recommended that LDL particle number is a more powerful tool to predict CVD events than LDL particle size or sdLDL [10]. Overall, measurement of sdLDL or LDL particle number together with the lipid profile (LDL-C, triglycerides, HDL-C and cholesterol) can be useful in the evaluation and management of CVD [10, 18].

One of the most important therapeutic targets for reducing CVD risk is to improve sdLDL-C levels [18]. Life-style changes emphasizing a healthy diet could play an important role in decreasing the atherogeneity of the lipid profile [19–21]. Functional food ingredients have attracted significant attention as natural products for the management of dyslipidemia and subsequently increase cardio-metabolic health [22–28]. Although nutraceuticals and herbal medicine have been previously studied as a non-pharmacological management of dyslipidemia [29–35], greater clarity on the effects of these bioactive natural compounds in improving sdLDL for the reduction in the relative risk of CVD is needed. To date, there is no study that has investigated the effect of these bioactive natural compounds on sdLDL levels. Therefore, the aim of the present review is to summarize the evidence accrued on the effect of special diet ingredients such as n-3 polyunsaturated fatty acids, nutraceuticals and herbal medicines on the levels of sdLDL, LDL particle number, and LDL particle size.

## Search strategy

The present narrative review was performed based on the PRISMA guidelines. The databases of PubMed, Web of Science, Google Scholar, and Scopus were searched up to August 2019 and limited to English language. Search strategies were included the following keywords in titles abstracts: (medicinal plants OR herbal bioactive OR bioactive natural compounds OR nutraceutical); AND (random OR randomized OR randomly OR randomization OR “randomized controlled trial” OR “randomized trial” OR “randomized study” OR “random number” OR placebo) AND (Small dense low-density lipoprotein OR sdLDL OR “LDL particle number” OR “LDL particle size” OR “LDL subfraction”).

### Phytosterols and vegetable oils

Phytosterols (plant sterols and stanol esters) have been reported to prevent the development of several chronic diseases including cancer, CVD and diabetes [36–39]. The results of previous meta-analyses indicated that phytosterols might improve lipid profile levels [40–42]. Phytosterols may decrease oxidized LDL and can be considered as an atherogenic modification for LDL. In a cross-over controlled trial, 25 healthy male volunteers were recruited to ingest 25 mL/d raw low-polyphenol-content olive oil (LPCOO; 366 mg/kg) or high-polyphenol-content olive oil (HPCOO; 2.7 mg/kg) for 3 weeks, separated by a two-week washout period. At the end of the study, in comparison to the baseline and LPCOO group, HPCOO intervention resulted in a significant reduction in small LDL particles. Conversely compared to baseline, LPCOO significantly increased small LDL particles [43]. In a trial study, 108 metabolic syndrome patients were randomized to receive one of two plant sterol-enriched yogurt mini drinks that contained 4 g phytosterols, or a yogurt beverage without phytosterols (control). After 2 months intervention, phytosterol supplementation significantly reduced sdLDL levels in the intervention group compared with the control group [44]. In another study, 59 children between 4.5–15.9 years of age were divided into 2 groups: (i) 25 subjects with a LDL-C ≥ (130 mg/dl to receive a yogurt-drink enriched with 2 g of plant sterols for 6 to 12 months (intervention group) or (ii) 34 subjects with a LDL-C < 130 mg/dl (control group). Results indicated that plant sterol consumption reduced sdLDL in the intervention group compared to the control group in which they were unchanged [45]. In a previous study, 56 healthy participants received a diet that contained high amounts of saturated fat for 2 weeks; and subsequently were randomized to one of three dietary intervention treatments; refined olive oil, rapeseed oil or sunflower oil as the principal source of fat for 4 weeks. The results showed that during the oil diet phase, LDL size significantly reduced though differences between groups were not significant and oil consumption did not significantly change LDL size variation [46]. In a recent report, 40 healthy subjects were randomly allocated to drink an inositol-enriched beverage (IEB) that contained inositols 2.23 g in 250 ml or a sucrose-sweetened beverage (SB) twice daily for 12 weeks. The study showed that those subjects in the IEB arm had a significant increase in LDL particle size compared to the SB group [42]. Shrestha et al. conducted a randomized, crossover study, in which 33 healthy adults were allocated to treatment cookies (7.68 g/d psyllium and 2.6 g/d plant sterols) and a placebo cookie (0 g psyllium and plant sterols) for 4 weeks (each separated by a 3-week washout period). At the end of the study, psyllium and plant sterol cookies reduced the medium-small LDL particles, and markedly increased mean and peak LDL particle sizes compared with placebo [47, 48]. In a randomized double-blind clinical trial, 54 adults with LDL-C level ≥ 3.33 mmol/L were randomized into two groups to receive four capsules of phytosterol esters (2.6 g/day phytosterol esters) or placebo (canola oil) per day for 12 weeks. At the end of the study the proportion of LDL, mean and peak LDL particle sizes did not differ between groups [49]. Matvienko et al. conducted a clinical trial study in which 34 men with moderate hypercholesterolemia and hypertriglyceridemia were randomly divided into two groups to consume ground beef with 2.7 g of phytosterols that included 67% sterol esters and 33% free sterols, or ground beef alone as the control group. After 4 weeks of intervention there were no differences between groups for small LDL particle and LDL peak particle size, though there was a trend for mean LDL particle number to decrease in the phytosterol treated group compared with the control group [50]. In another trial, 28 participants with familial combined hyperlipidemia (FCHL) and elevated triacylglycerols consumed 2.5 g plant stanol ester-enriched margarine per day or margarine without stanols as control for 3 weeks. No cholesterol-lowering drugs were used 2 weeks before and during the study. At the end of the study after stanol supplementation the number of total LDL particles decreased, but sdLDL particles did not differ compared with baseline [51]. Utarwuthipong et al. [52] also reported a study of 16 hyperlipidaemic women who were recommended a National Cholesterol Expert Panel diet (55% carbohydrate, 15% protein, 30% fat, < 300 mg/day of cholesterol) as a run in baseline diet for 8 weeks. All participants were subsequently randomized into four treatment groups to take either diet plus SBO (soybean oil, 20% of total energy), or diet plus RBO (rice bran oil, 20% of total energy) or diet plus PO (palm oil, 20% of total energy) or diet plus RBO/PO (20% of total energy as mixture of (3:1) RBO/PO). Every 10 weeks, they were crossed over to another group and therefore they participated for a total of 48 weeks. The sdLDL particles were measured via a sequential ultracentrifugation technique. Results showed that SBO and RBO/PO significantly reduced sdLDL-cholesterol levels, though PO significantly increased sdLDL-cholesterol levels and RBO did not differ compared to the control diet (Table 1). Overall, in almost all of the studies reviewed here, vegetable oils and phytosterols showed promising effects on sdLDL-cholesterol levels, and LDL-particle size. In 4 studies out of 6, sdLDL was decreased after the intervention with vegetable oils and phytosterols, and in 2 studies out of 4, LDL particle size increased after the intervention. In the 2 studies that sdLDL did not decrease and in one study that LDL particle size did not change, LDL particle number significantly decreased.

**Table 1:** The effect of phytosterols and vegetable oils on plasma concentration of small dense low density lipoprotein (LDL), LDL particle number, and LDL particle size

Author, Year	Intervention	Dose per day	Treatment duration	Subjects	Method of assessment	Main outcomes	Final effects of specific diet ingredients or nutraceuticals on LDL (number, size and concentration)
Hernáez et al. 2015 [43]	Olive oil	25 mL/d raw LPCOO; 366 mg/kg or HPCOO; 2.7 mg/kg)	3 weeks	25 healthy volunteer men	NMR spectroscopy	HPCOO significantly reduced small LDL particles (− 15.3%), though LPCOO significantly increased small LDL particles (+ 13.6%), differences between groups were significant	Small dense LDL **↓**
Sialvera et al. 2010 [44]	Phytosterols	4 g/day	2 months	108 metabolic syndrome patients	sLDL e EX “SEIKEN”	Phytosterols supplementation significantly reduced small dense LDL levels (−3.9 mg/dl) in the intervention group compared with the control group	Small dense LDL **↓**
Garoufi et al. 2014 [45]	Plant sterols	2 g/day	6–12 months	59 hypercholesterolemic and normal children (4.5–15.9 years)	kit (sLDL-EX “SEIKEN”	Plant sterols consumption considerably reduced sdLDL in the intervention group though levels remained higher than control group	Small dense LDL **↓**
Kratz et al. 2002 [46]	Vegetable oils	Refined olive oil, rapeseed oil, sunflower oil	4 weeks	56 healthy adults	PAGE	In response to vegetable oils, LDL size significantly reduced (−0.36 nm) thought differences between groups were not significant. Furthermore, oil consumption did not significantly change LDL size variation	LDL particle size —
Banuls et al. 2015 [42]	IEB, SB	4.45 g/day	12 weeks	40 healthy adults	PAGE	IEB led to significant elevation in LDL particle size (0.13 nm) compared with baseline and with SB.	LDL particle size **↑**
Shrestha et al. 2006 [48] 2007 [47]	Treatment cookies, Placebo cookies	Treatment cookies; 7.68 g/d psyllium and 2.6 g/d plant sterols, or Placebo cookies; 0 g psyllium and plant sterols	3 months	33 healthy adults	Nongradient, high-resolution PAGE and NMR	psyllium and plant sterols were reduced the medium-small LDL particles (− 18.9 ± 0.7 nmol/L), and considerably increased LDL mean size (+ 0.11 ± 0.04 nm) and LDL peak size (+ 0.2 ± 0.15 nm) in compared with placebo.	Medium-Small LDL particles **↓** LDL particle size **↑**
Earnest et al. 2007 [49]	Phytosterol esters, canola oil (placebo)	2.6 g/day	12 weeks	54 adults with LDL-C level ≥ 3.33 mmol/L	Relative migration of four plasma standards of known diameter was used to estimate LDL particle size. The estimated diameter for the major peak in each scan was identified as the LDL peak particle size	Proportion of LDL, mean and peak LDL particle sizes did not change in both groups.	LDL particle size —
Matvienko et al. 2002 [50]	Ground beef with phytosterols, Control (ground beef alone)	2.7 g of phytosterols	4 weeks	34 men with elevated plasma TC and LDL cholesterol	Nondenaturing PAGE and NMR	No significant change in small LDL particle and LDL peak particle size. However, mean LDL particle number decreased than control.	Small dense LDL — LDL particle size — LDL particle number **↓**
Theuwissen et al. 2009 [51]	Stanol supplementation	2.5 g/day of plant stanols	3 weeks	28 adults with elevated triacylglycerols	NMR by Liposcience	After stanol supplementation the number of total LDL particles decreased, but sdLDL particles did not significantly change compared with baseline.	Small dense LDL — LDL particle number **↓**
Utarwuthipong et al. 2009 [52]	SBO; RBO; PO; mixture of (3:1) RBO/PO	20% of total energy as SBO; 20% of total energy as RBO; 20% of total energy as PO; 20% of total energy as mixture of (3:1) RBO/PO.	10 weeks	16 hypercholesterolemia women	Sequential ultracentrifugation technique	SBO and RBO/PO significantly reduced sdLDL-cholesterol level (− 10%) and (− 5%) respectively, though PO significantly increased sdLDL-cholesterol level (+ 5%) and RBO was not significantly varied, differences between groups were significant.	Small dense LDL **↓**

*sdLDL* small dense Low-Density Lipoprotein, *LDL-C* Low-density lipoprotein cholesterol, *TC* total cholesterol, *LPCOO* low-polyphenol-content olive oil, *HPCOO* high-polyphenol-content olive oil, *PAGE* Polyacrylamide gradient gel electrophoresis, *NMR* Nuclear magnetic resonance, *IEB* Inositol-enriched beverage, *SB* Sucrose-sweetened beverage, *SBO* Soybean oil, *RBO* Rice bran oil, *PO* Palm oil, *mg/dl* milligrams per decilitre, *nmol/l* nanomoles per litre, *nm* nanometer

### Eicosapentaenoic acid and docosahexaenoic acid

Eicosapentaenoic acid (EPA) and docosahexaenoic acid (DHA) derived from alpha-linolenic acid (ALA) are one of the family of essential fatty acids derived from the diet, as they cannot be made endogenously, that are involved in cell membrane stability [53]. It is suggested that EPA and DHA may have beneficial effects on the treatment of NAFLD [54], diabetes [55] and cardiovascular disease [56, 57]. However, there has been concern whether n-3 fatty-acid enhances the susceptibility of LDL to oxidation, though the data remains inconclusive [58–60]. In a comparative study, 5 g/day fish oil supplement containing 1.9–2.2 g EPA and 1.1 g DHA were given to 210 healthy adults for 6 weeks. At post intervention, there was no significant change in LDL particle size compared to baseline [61]. Oelrich et al. conducted a clinical trial in which 60 subjects with moderate hypertriglyceridemia randomly were divided into 4 groups to consume 4 g/day fish oil supplementation in three formulations ((i) 90% TG formulation, (ii) 60% TG formulation, or (iii) 0% TG ethyl esters) with a soy oil supplement as the control group. LDL particles were assessed by gradient gel electrophoresis. After 12 weeks intervention, in all fish oil groups, four particle sizes in terms of LDL_1_, LDL_2_, and LDL_3_ significantly increased in comparison to the baseline, though the concentration of LDL_4_ did not differ [62]. In a double blind, placebo controlled trial, 42 diabetic patients with moderate hypercholesterolemia were treated with corn oil capsules (4 g/ day) for 4 weeks, then they were randomly divided into two groups to consumed fish oil tablets (2.6 g EPA and DHA, plus 13.4 mg vitamin E), or corn oil tablets (1 g corn oil plus 13.4 mg vitamin E) four times a day. Results of this study showed that sdLDL particles did not significantly change in the EPA/DHA intervention group compared with control group [63]. In a randomized crossover trial, 20 hypertensive adults ingested four capsules of fish oil (4 g per day) or corn oil tablet as a placebo for 6 weeks that resulted in an increase in LDL size (+ 0.16 nm) in the fish oil group [64]. In another clinical trial, 121 healthy individuals with normal lipid profiles were randomly assigned to take 600 mg/day EPA, 1800 mg/day EPA, 600 mg/day DHA or 6 g/day olive oil that was used as the comparator. After 6 weeks of supplementation, sdLDL was unchanged between and within the four groups [65]. Satoh et al. [66] randomized 44 obese participants with ≥2 risk factors of metabolic syndrome into two groups; diet plus 1.8 g/day EPA (intervention group) or diet alone (control group) for 3 months. The diet was that of the Japan Atherosclerosis Society Guidelines and included 60% of total energy from carbohydrates, 15–20% from protein and 20–25% from fat by following the ratio 3:4:3 as polyunsaturated, monounsaturated and saturated fatty acids, respectively. In addition, the daily diet was based on 25 kcal/kg of ideal body weight. Results indicated that sdLDL and sdLDL proportion significantly decreased in the EPA group compared with baseline, but did not differ between the two groups. In another study, 59 overweight adults with mild hypercholesterolemia were randomized to consume 4 g EPA every day, or DHA or olive oil as the comparative control for 6 weeks. There was no significant difference in LDL particle size between and within groups; however, after adjustment for baseline values, LDL particle size significantly increased (0.25 ± 0.08 nm) in the DHA group compared with olive oil (placebo) [67]. In a study of 34 healthy or mildly hyperlipidemic men who were randomized to two groups: (i) 7.5 g/day DHA (~ 3 g/day DHA), (ii) 7.5 g/day olive oil (comparator). After 90 days of intervention, participants who consumed DHA had fewer small dense LDL particles though this did not differ compared to the control, though there was a significant increase in mean LDL particle compared with baseline [68]. In another clinical trial, 57 subjects with below average levels of HDL were given either DHA capsules (1.52 g DHA per day) or olive oil capsules as a control group for 6 weeks. Results revealed that DHA had no effect on sdLDL though the percentage of cholesterol in the small dense LDL was significantly decreased by DHA in comparison with the control group [69]. In another crossover study, 15 healthy adult males were randomized into 2 groups to receive 10 g flaxseed oil (5.49 g of α-Linolenic acid) or 10 g corn oil (0.09 g of α-Linolenic acid) as placebo for 12 weeks. Results showed that in the intervention group, the concentration of sd-LDL significantly reduced after 4 and 12 weeks of flaxseed oil supplementation compared with baseline (− 25.8% and − 21.2% respectively). In addition, sd-LDL concentrations decreased significantly compared with the control group after 4 weeks [70]. In a study of 56 patients without known coronary heart disease, they were assigned to receive 5.2 g flaxseed oil (3 g/d of ALA), or 5.2 g of olive oil as control per day for 26 weeks. At the end of the study there was no effect of flaxseed oil on atherogenic LDL subfractions (LDL_3_ and LDL_4_); however, flaxseed oil significantly increased the less atherogenic LDL subfractions (LDL_1_ and LDL_2_) as compared to the control olive oil [71] (Table 2). Overall, in 3 out of 5 studies reviewed here, LDL particle size significantly increased after the intervention; however, in 2 studies out of 6 studies sdLDL was significantly decreased though other studies did not find any significant changes.

**Table 2:** The effect of eicosapentaenoic acid and docosahexaenoic acid (EPA & DHA) on plasma concentration of small dense low density lipoprotein (LDL), LDL particle number, and LDL particle size

Author, Year	Intervention	Dose per day	Treatment duration	Subjects	Method of assessment	Main outcomes	Final effects of specific diet ingredients or nutraceuticals on LDL (number, size and concentration)
Ouellette et al. 2014 [61]	Fish oil supplementation	5 g/day	6 weeks	210 healthy adults	2–16% PAGE	The n-3 PUFA supplementation had no effect on plasma LDL-C concentrations and LDL particle size	LDL particle size —
Oelrich et al. 2013 [62]	Fish oil supplementation, Soy oil supplement (placebo)	4 g/day (800 mg EPA and DHA)	12 weeks	60 hypertriglyceridemic adults	Gradient gel electrophoresis	In comparison to the baseline, four particle sizes in terms of LDL_1_ (+ 20 ± 5%), LDL_2_ (+ 64 ± 13%), and LDL_3_ (+ 26 ± 6%) significantly increased in all fish oil groups.	LDL particle size **↑**
Petersen et al. 2002 [63]	Fish oil supplementation, Corn oil supplement (placebo)	4 g/day (2.6 g EPA and DHA + 13.4 mg vitamin E)	8 weeks	42 diabetic patients with moderate hypertriglyceridemia	Ultracentrifugation	Small dense LDL particles did not significantly change compared with control group.	Small dense LDL
Suzukawa et al. 1995 [64]	Fish oil, Corn oil (placebo)	4 g/day (3.4 g of n-3)	6 weeks	20 hypertensive adults	ND	Fish oil increased LDL size (+ 0.16 nm) compared to the baseline.	LDL particle size **↑**
Asztalos et al. 2016 [65]	EPA, DHA, Olive oil (placebo)	EPA 600 mg/day EPA 1800 mg/day DHA 600 mg/day Olive oil placebo 6 g/day	6 weeks	121 healthy individuals with normal lipid profiles	Denka-Seiken Corporation (for LDL-C and sdLDL-C, Tokyo, Japan)	In all 4 groups no significant change in sdLDL-C (*p* = 0.82)	Small dense LDL —
Satoh et al. 2007 [66]	EPA	1.8 g/d	3 months	44 obese patients with metabolic syndrome	The Quantimetrix Lipoprotein LDL system (LDL3–7:sdLDL)	sdLDL (− 5 mmol/l) and sdLDL proportion (− 1.27%) significantly decreased in EPA group compared with baseline but no significant differences between the two groups.	Small dense LDL **↓**
Mori et al. 1999 [67]	EPA, DHA, Olive oil (placebo)	4 g/d	6 weeks	59 overweight adults with mildly hypercholesterolemic	Using commercially available 3–13% nondenaturing native gels	There was no statistically significant difference in LDL particle size between and within groups.	LDL particle size —
Kelley et al. 2007 [68]	DHA, Olive oil (placebo)	7.5 g/d DHA oil (~ 3 g/d DHA), or 7.5 g/d olive oil	90 days	34 healthy or mildly hyperlipidemic men	NMR	Participants who consumed DHA had lower the number of small dense LDL particles (21%) whereas the change was not statistically significant compared to the placebo. Mean LDL particle size significantly increased (0.6 nm or 3%) compared with baseline.	LDL particle size **↑** LDL particle number **↓**
Maki et al. 2005 [69]	DHA, Olive oil (placebo)	1.52 g/day DHA	6 weeks	57 subjects with below average levels of HDL	Vertical Auto Profile II (VAP-II)	DHA had no effect on small dense LDL. In addition, the percentage of cholesterol concentration in small dense LDL was significantly reduced in the DHA group than the control (− 9.7 vs. -3.0%).	Small dense LDL — Cholesterol concentration in small dense LDL **↓**
Kawakami et al. 2015 [70]	FO, Corn oil (placebo)	10 g FO (5.49 g of ALA), 10 g corn oil (0.09 g of ALA)	12 weeks	15 healthy males	sd-LDL-EX “SEIKEN”	In the intervention group, concentration of sd-LDL significantly reduced after 4 (− 25.8%) and 12 weeks (− 21.2%) in compared with the baseline. In addition, sd-LDL concentrations significant decreased than placebo after 4 weeks.	Small dense LDL **↓**
Harper et al. 2006 [71]	FO supplementation, Olive oil capsules (placebo)	5.2 g/d FO (3 g/d of ALA), Or 5.2 g of olive oil	26 weeks	56 patients without known coronary heart disease	Ultracentrifugal separation by enzymatic determination of cholesterol with 400 sequential spectrophotometric measurements	FO had no effect on atherogenic LDL subfractions (LDL_3_ and LDL_4_). However, FO significantly increased on less atherogenic LDL subfractions (LDL_1_ and LDL_2_) as compared to the olive oil (+ 0.08 and + 0.01 mmol/l) and the baseline (+ 0.06 and + 0.1 mmol/l).	Small dense LDL — LDL subfractions (LDL1 and LDL2) **↓**

*sdLDL* small dense Low-Density Lipoprotein, *LDL-C* Low-density lipoprotein cholesterol, High Density Lipoprotein Cholesterol, *PUFA* polyunsaturated fatty acid, *ALA* α-Linolenic acid, *EPA* Eicosapentaenoic acid, *DHA* Docosahexaenoic acid, *NMR* Nuclear magnetic resonance, *FO* Flaxseed oil, *mg* milligrams, *mmol/l* millimoles per litre, *nm* nanometer

### Fruits

Fruits are a rich source of flavonoids, polyphenols, fiber and have antioxidant properties that have been reported to have beneficial effects on various metabolic disorders [72–75]. In a randomized 3-period crossover study design, 45 overweight participants with baseline LDL entered into a 2-week run-in period taking an average American diet (51% carbohydrate, 16% protein, 34% fat, 13% saturated fat). Following a run-in period, participants were randomly allocated to receive either an avocado diet (one fresh Hass avocado contained 136 g fruit pulp and 13 g monounsaturated fatty acids (MUFA)) or a low-fat diet (59% carbohydrate, 16% protein, 24% fat, 7% saturated fat contained grains instead of SFA) or a moderate-fat diet (49% carbohydrate, 16% protein, 34% fat, 6% saturated fat that contained high oleic acid oils, sunflower oil and canola oil). After a 5 week period, subjects were washed out over a 2-week period and crossed over for each of the interventions. Results showed that LDL particle number and small dense LDL cholesterol number were significantly lower following the AV diet compared with baseline. LDL particle size was reduced in all diets, but compared with the LF diet the AV diet led to a significant rise in LDL particle size [76]. In a controlled, 3-arm, crossover study, 31 overweight or obese subjects with a fasting glucose that ranged 5.0–6.4 mmol/L were randomized to either Half-Avocado group who received 68 g/d fresh avocado or Whole-Avocado contained 136 g avocado per day or control group (without any avocado). After 6 h following ingestion, lipoprotein particles were measured by NMR spectra of frozen plasma. Those that consumed a whole avocado showed a significant reduction in sdLDL particles compared with the control [77]. In a clinical trial, 27 subjects with metabolic syndrome were divided into either strawberry or control groups. The strawberry group received 2 cups of strawberry beverage plus 2 cups of water while the control group were given only 4 cups of water per day Following strawberry supplementation there was a significant reduction in the concentration of small LDL particles compared to control; however, LDL mean particle size was unchanged between the two groups [78]. In a recent study, 60 subjects with elevated lipid profiles were randomly assigned to either low-dose freeze-dried strawberries (FDS) [LD-FDS; 25 g strawberries with 2 cups of water] or low-dose control [LD-C; 4 g of fiber plus 20 g of cane sugar with 2 cups of water] or high-dose FDS [HD-FDS; 50 g strawberries with 2 cups of water] or 4) high-dose control [HD-C; 8 g of fiber plus 36 g of cane sugar with 2 cups of water] for 12 weeks. Results showed that, the HD-FDS diet significantly decreased small LDL particles compared with the HD-C diet, and this differed significantly compared to the LD-FDS diet [79]. In a randomized cross-over design, 20 obese adults were randomized into two groups and followed for 3 weeks. The intervention group received 80 g strawberry powder four times daily, the control group were given a strawberry flavoured preparation dyed with red food color. The results showed that LDL particle size significantly increased in the intervention group in comparison to the control group; however, the strawberry diets had no effect on the concentrations of small LDL [80]. In an another cross-over study, 24 obese adults were randomized to either the intervention group (46 g freeze-dried grape powder with 240 ml of water two times a day) or the control group (46 g fructose, glucose, organic acids and fiber with 240 ml of water two times a day). After a 3 week intervention period separated by 3 week washout periods, LDL particle size and concentrations of small LDL did not change in the study groups [81]. A prospective study evaluated the effectiveness of Bergamot supplementation for the treatment of dyslipidemic patients. A total 80 participants with moderate hypercholesterolemia received 150 mg/day Bergavit® (Bergamot flavonoids containing 16% of neoeriocitrin, 47% of neohesperidin and 37% of naringin) for 6 months. After 6 months, Bergavit® supplementation significantly reduced sdLDL-3, − 4, and 5 particles compared to baseline [82]. In another study, 107 subjects with metabolic syndrome and NAFLD were given 1300 mg/d Bergamot polyphenolic fraction (BPF) in a capsule which contained flavonoids composed of 370 ppm of neoeriocitrin, 520 ppm of naringin, and 310 ppm of neohesperidin. The results showed that sdLDL particles were significantly reduced after the 120 day intervention with BFP compared to baseline [83] (Table 3). Overall, in 6 out of 7 studies sdLDL was significantly reduced by fruit consumption, and in 2 out of 4 studies LDL particle size increased. In one study, in addition to the significant decrease in sdLDL, LDL particle number was also decreased.

**Table 3:** The effect of fruits on plasma concentration of small dense low density lipoprotein (LDL), LDL particle number, and LDL particle size

Author, Year	Intervention	Dose per day	Treatment duration	Subjects	Method of assessment	Main outcomes	Final effects of specific diet ingredients or nutraceuticals on LDL (number, size and concentration)
Galletti et al. 2019 [84]	Armolipid Plus**®** (AP)	AP; berberine 500 mg, red yeast rice, monacolin K 3 mg and policosanol 10 mg	24 weeks	147 metabolic syndrome patients	Electrophoretic mobility	Significant elevations in sdLDL-C size (+ 1 Å) after taking AP tablet compared with baseline.	LDL particle size **↑**
Wang et al. 2015 [76]	Avocado diet (AV) Lower-fat diet (LF) Moderate-fat diet (MF)	AV; fresh Hass avocado (136 g/d) or LF; 59% carbohydrate, 16% protein, 24% fat, 7% saturated fat or MF; 49% carbohydrate, 16% protein, 34% fat, 6% saturated fat	5 weeks	45 overweight or obese subjects with baseline LDL-C	NMR spectroscopy	LDL particle number (− 80.1 nmol/L), small dense LDL cholesterol (− 4.1 mg/dL) were significantly lower in AV diet compared with baseline. LDL particle size was reduced in all diets, LF (− 0.24 nm), MF (− 0.21 nm), and AV (− 0.12 nm) but compared with the LF diet, AV diet led to significant rise in LDL particle size (+ 0.12 nm).	Small dense LDL **↓** LDL particle number **↓**
Park et al. 2018 [77]	Avocado (Half-A), Avocado (Whole-A), Control	Half-A; 68 g, Whole-A; 136 g, Control; 0 g	6 h postprandial	31 overweight or obese subjects	NMR	After consumed whole avocado, small dense LDL particles significantly reduced (36.8 nmol/l) in compared with control.	Small dense LDL **↓**
Basu et al. 2010 [78]	Strawberries	50 g freeze-dried strawberries or 3 cups fresh strawberries	8 weeks	27 subjects with metabolic syndrome	NMR	Strawberry supplementation led to significant reduction in concentration of small LDL particles (14%), this change was significant differences between control and intervention groups. LDL mean particle size was not significantly varied between two groups.	Small dense LDL **↓** LDL particle size —
Basu et al. 2014 [79]	LD-FDS, LD-C, HD-FDS, HD-C	LD-FDS; 25 g/d strawberries, LD-C; 4 g fiber + 20 g cane sugar HD-FDS; 50 g/d strawberries, HD-C; 8 g fiber + 36 g cane sugar	12 weeks	60 subjects with elevated lipid profiles	NMR	HD-FDS diet significantly decreased small LDL particles (− 323 ± 16 nmol/l) compared with HD-C diet. Furthermore, the change was statistically significant compared to the LD-FDS.	Small dense LDL **↓**
Zunino et al. 2011 [80]	Strawberry powder	320 g/day frozen strawberries	3 weeks	20 obese subjects	NMR	LDL particle size significantly increased (+ 0.43 ± 0.08 nm) in intervention group in comparison to the control group.	LDL particle size **↑**
Zunino et al. 2014 [81]	Grape powder	46 g grape powder	3 weeks	24 obese subjects	NMR	Grape diets had no effect on small LDL and LDL size.	Small dense LDL — LDL particle size —
Toth et al. 2016 [82]	Bergavit®	150 mg/d of flavonoids, with 16% of neoeriocitrin, 47% of neohesperidin and 37% of naringin	6 months	80 subjects with moderate hypercholesterolemia	Gel electrophoresis	Bergavit supplementation significantly reduced small dense LDL-3, − 4, and 5 particles (− 38, − 53%, − 67%, respectively) than baseline.	Small dense LDL **↓**
Gliozzi et al. 2014 [83]	BPF	1300 mg/day	120 days	107 subjects with metabolic syndrome and NAFLD	NMR Spectroscopy	Small dense LDL particles significantly reduced (− 374 ± 7 nmol/l) after 120 days intervention than baseline.	Small dense LDL **↓**

*sdLDL* small dense Low-Density Lipoprotein, *HDL-C* High Density Lipoprotein Cholesterol, *LDL-C* Low-density lipoprotein cholesterol, *NAFLD* Non-alcoholic Fatty Liver Disease, *NMR* Nuclear magnetic resonance, *FO* Flaxseed oil, *AP* Armolipid Plus**®**, *AV* Avocado diet, *LF* Lower-fat diet, *MF* Moderate-fat diet, *LD-FDS* Low-dose freeze-dried strawberries, *LD-C* Low-dose control, *HD-FDS* High-dose freeze-dried strawberries, *HD-C* High-dose control, *BPF* Bergamot polyphenolic fraction, *nmol/l*. nanomoles per litre, *nm* nanometer, *Å* Angstrom

### Nuts

Nuts contain unsaturated fats, soluble fiber, antioxidants, and phytosterols that have potentially beneficial effects on serum lipids, blood pressure, and on inflammation [85]. A meta-analysis of clinical trials concluded that nut intake led to a significant beneficial effect on triglycerides, total cholesterol, LDL, and Apo B [86]. Furthermore, the findings of a previous systematic review showed that nuts especially almonds, pistachios, brazil nuts, peanuts, and hazelnuts may provide protection from oxidation LDL to due to the presence of bioactive antioxidant compounds in nuts. However, in some nuts such as walnuts due to their high content of unsaturated fatty acids this may increase the sensitivity to oxidation [87, 88]. In a randomized crossover study, a pistachio-supplemented diet (50% carbohydrates, 33% fat, included 57 g/day of pistachios) and control diet (55% carbohydrates, 30% fat) was administrated to 54 prediabetic patients for 2 weeks, with a 2-week washout period between interventions. The results showed that the small LDL number was significantly decreased in the pistachio-supplemented diet compared to the control diet, whereas the LDL particle size did not differ between the groups [89]. Oliver Chen et al. conducted a randomized, crossover intervention, in which 45 patients with coronary artery disease were given a control diet without nuts (National Cholesterol Education Program (NCEP) diet) or an intervention diet of 85 g per day of almonds added to the NCEP diet for a 6 week period (each separated by a 4 week washout period). At the end of the study, no significant effect was observed on small density LDL-C levels following the consumption of almonds [90]. In a single-intervention study design, 21 healthy adults were given a hazelnut-enriched diet of 1 g/kg/day of hazelnuts for 4 weeks. After 30 days, small LDL levels were significantly decreased compared with baseline [88]. In another study, 18 hyperlipidemic adults (13 postmenopausal women and 5 men), sequentially entered 4 stages of the interventional diet; 1) a habitual diet, 2) a habitual diet plus walnuts, 3) a low-fat diet, and 4) a low-fat diet plus walnuts. Each diet was undertaken for 4 to 6 weeks. At the end of the study, the results showed that all four diets did not affect LDL particle size; however, walnut supplementation (48 g walnuts/8460 kJ energy intake) led to a significant reduction in the distribution of cholesterol in the small LDL compared with a habitual diet [91]. In a randomized 4-period crossover study, 48 overweight adults were randomized to four groups to consume 42.5 g/d of almonds (ALD), 18 g/d of cocoa powder and 43 g/d of dark chocolate (CHOC), a combination of almonds, cocoa, and chocolate (CHOC+ALD), or an average American diet (AAD) as the control group. Each intervention was over a 4-week period with a 2-week washout periods between each. Results showed that, the concentration of sdLDL particles were significantly reduced in (CHOC+ALD) group compared with the control group (AAD), suggesting that the combination of almonds, cocoa, and chocolate could have a benefit to improve the risk of coronary heart disease [92]. In the PREDIMED trial, 169 diabetics, hyperlipidemic or hypertensive were randomly divided into those taking a Mediterranean diet (MeDiet) + extra-virgin olive oil (EVOO; 1 L/weekl) or MeDiet plus mixed nuts containing15 g walnuts, 7.5 g hazelnuts and 7.5 g almonds, or control group who were advised on a low-fat diet, for 1 year. At the end of the study, compared with the baseline, MeDiet + nuts led to a significant reduction in medium-small LDL (10%), very small LDL (11%) and a significant rise in LDL size (+ 0.2 nm). In addition, sdLDL particle levels decreased significantly in the MeDiet + nuts group compared with the control group, and the increase in the LDL size change was statistically significant compared to the other groups [93]. In a three-period, cross-over study, 28 healthy adults were assigned into three groups of; a 1 serving of pistachio per day (1 PD) diet that contained 30% energy from total fat (TF), 8% from SFA, and 10% from pistachios (one serving of pistachios/d or 32–63 g/d), a 2PD diet that contained 34% energy from TF, 8% from SFA, and 20% from pistachios (two serving of pistachios/d or 63–126 g/d), or a control diet that contained 25% energy from TF and 8% from SFA (lower-fat diet). Each of the diets was conducted over a 4-week period with a 2-week washout period between the diets. The results showed that the 2PD treated group had a significant reduction in sdLDL in compared to both the 1PD and control groups [94] (Table 4). Overall, in 5 out of 6 studies consumption of nuts significantly reduced sdLDL. LDL particle size was increased in 1 out of 3 studies, in 1 study the distribution of cholesterol in the small LDL was decreased.

**Table 4:** The effect of nuts on plasma concentration of small dense low density lipoprotein (LDL), LDL particle number, and LDL particle size

Author, Year	Intervention	Dose per day	Treatment duration	Subjects	Method of assessment	Main outcomes	Final effects of specific diet ingredients or nutraceuticals on LDL (number, size and concentration)
Hernández-Alonso et al. 2015 [89]	PD, control diet	PD; 50% carbohydrates, 33% fat, including 57 g/d of pistachios, Control diet, 55% carbohydrates, 30% fat	2 weeks	54 prediabetic patients	NMR	Small LDL particle was significantly decreased in PD compared to the control diet (change: − 28.07 nM vs + 16.49 nM), whereas the LDL particle size did not significantly change between two groups.	Small dense LDL **↓** LDL particle size —
Chen et al. 2015 [90]	Almonds	85 g/day	6 weeks	45 patients with coronary artery disease	Olympus AU400 auomated analyzer	No significant effect was observed on small density LDL-C following consumption of almonds.	Small dense LDL —
Yücesan et al. 2010 [88]	Hazelnut	1 g/kg/day	4 weeks	21 healthy adults	ND	After 30 days, small LDL was significantly decreased (− 0.04 mmol/l) as compared with baseline.	Small dense LDL**↓**
Almario et al. 2001 [91]	HD, HD + W, LFD, LFD + W	48 g walnuts/8460 kJ energy intake	6 weeks	18 hyperhypidemic adults	NMR	All four diets did not influence in LDL particle size. However, walnut supplementation led to a significant reduction in distribution of cholesterol in the small LDL compared with HD (− 12.7%).	LDL particle size — distribution of cholesterol in the small LDL **↓**
Lee et al. [92]	AAD, ALD, CHOC, CHOC+ALD	ADD; no treatment foods or ALD; 42.5 g/d of almonds or CHOC; 18 g/d of cocoa powder and 43 g/d of dark chocolate	22 weeks	48 obese or overweight adults	ND	Consumption of dark chocolate, cocoa, and almonds significantly reduced small LDL particles (− 6.7 mg/dl) compared with the AAD.	Small dense LDL **↓**
Damasceno et al. 2013 [93]	MeDiet + EVOO, MeDiet + nuts, Control diet	(MeDiet + EVOO); 1 L/week extra-virgin olive oil, or (MeDiet + nuts); 30 g/day of mixed nuts (15 g walnuts, 7.5 g hazelnuts and 7.5 g almonds), or Control diet; advised on a low-fat diet	1 year	169 diabetics, hyperlipidemic or hypertensive	NMR spectroscopy	Medium-small LDL (10%), and very small LDL (11%) decreased significantly in (MeDiet + nuts) group compare with baseline. However, small dense LDL particles decreased significantly in MeDiet + nuts group compare with control group. LDL size increased significantly in MeDiet + nuts group (+ 0.2 nm) compared with baseline and the change was statistically significant compared to the other groups.	Small dense LDL**↓** LDL particle size **↑**
Holligan et al. 2014 [94]	1PD, 2PD, Control	1PD; one serving (32–63 g) of pistachios per day (10% energy from pistachios) (30% total fat and 8% saturated fatty acids), or 2PD; two servings (63–126 g) of pistachios per day (20% energy from pistachios) (34% total fat and 8% saturated fatty acids), or Control; lower-fat diet (25% total fat and 8% saturated fatty acids)	4 weeks	28 healthy adults	removed from the plasma by filtration after the formation of aggregates with a polyanion and divalent cation-based reagent, and sdLDL levels were then determined using a Cobas 6000 analyser, with reagents obtained	The 2PD treated group revealed significantly reductions in small dense LDL in compared with 1PD (− 0.14 mmol/l) and control group (− 0.21 mmol/l).	Small dense LDL **↓**

*sdLDL* small dense Low-Density Lipoprotein, *LDL-C* Low-density lipoprotein cholesterol, *PD* Pistachio-supplemented diet, *HD* Habitual diet, *HD + W* habitual diet plus walnuts, *LFD* low-fat diet, *LFD + W* low-fat diet plus walnuts, *AAD* Average American diet, *ALD* Almond diet, *CHOC* chocolate diet, *MeDiet + EVOO* Mediterranean diets with extra-virgin olive oil supplementation, *MeDiet + nuts* Mediterranean diets with nuts supplementation, *NMR* Nuclear magnetic resonance, *mg/dl* milligrams per decilitre, *nmol/l.* nanomoles per litre, *mmol/l* millimoles per litre, *nm* nanometer, *ND* no data

### Curcumin

Curcumin is an active ingredient of turmeric spices (*Curcuma longa* L.) and has been used as a food spice and herbal remedy for centuries in different traditional medicine systems [95]. Because of its highly bioactive compounds it has been reported to have several pharmacological effects including anti-inflammatory, anti-tumor, lipid-modifying, antioxidant, anti-steatotic, anti-fibrotic, cardioprotective and antithrombotic activities [96–105]. The anti-atherosclerotic and cardioprotective effects are thought to be mediated through reduced LDL-oxidation [106, 107]. In a recent report, 117 metabolic syndrome patients were asked to consume 1000 mg/day curcuminoids plus 5 mg piperine (intervention group) or 5 mg piperine as the comparative control for 8 weeks. At the end of the study, sdLDL levels significantly decreased in the intervention group compared with baseline and between the two groups [97]. In a randomized crossover study, 30 obese participants with hyperlipidemia who were treatment naïve were randomized into either the curcuminoids group (1000 mg/day + 5 mg piperine) or the placebo group (5 mg piperine) for a 4-week period, with a 2-week washout period and then crossed over for a further 4 week period. At the end of the trial, no significant change in sdLDL concentrations were observed after taking the curcumin supplement compared with placebo [108] (Table 5). In 2 studies reviewed here sdLDL was decreased in 1 study after supplementation with curcumin.

**Table 5:** The effect of curcumin on plasma concentration of small dense low density lipoprotein (LDL), LDL particle number, and LDL particle size

Author, Year	Intervention	Dose per day	Treatment duration	Subjects	Method of assessment	Main outcomes	Final effects of specific diet ingredients or nutraceuticals on LDL (number, size and concentration)
Panahi et al. 2014 [97]	Curcuminoids- piperine	1000 mg/day + 10 mg piperine	8 weeks	117 metabolic syndrome patients	Immuno turbidimetric (sdLDL) methods with commercial kits	sdLDL levels significantly decreased in intervention group compared with baseline (− 2.73) and significant differences were observed between the two groups.	Small dense LDL**↓**
Moohebati et al. 2014 [108]	Curcuminoids- piperine	1000 mg/day + 5 mg piperine	4 weeks + 2 week washout phase	30 obese dyslipidemic subjects	Immunoturbidimetric assay	No significant change in sdLDL concentrations was observed after taking curcumin supplementation compared with placebo and baseline.	Small dense LDL —

*sdLDL* small dense Low-Density Lipoprotein, *LDL-C* Low-density lipoprotein cholesterol, *mg* milligrams

### Other nutraceuticals

#### Oolong tea

Oolong tea is partially fermented tea extracted from the leaves of *Camellia sinensis*, and contains high levels of polyphenolic compounds. It has been shown to improve the lipid profile especially effective in lowering cholesterol [109, 110]. In one clinical trial study, 12 patients with previous myocardial infarction and 10 patients with stable angina pectoris were divided into two groups and asked to drink 1000 ml/day (two bags of tea) oolong tea, or water as a control group for 4 weeks. At the endo of the study, a significant increase in plasma LDL particle size was observed in the oolong tea group compared with baseline [111].

#### Brown rice

Brown rice, as whole grain rice, is nutrient-rich including fiber, phytic acids, vitamins B and E, α-tocopherol, γ -oryzanol and γ -amino butyric acid (GABA) [112]. In a randomized controlled trial, 42 prediabetic, overweight patients were randomly assigned into 400 g partially-abraded brown rice (PABR) or white rice (WR) daily for 12 weeks. The results showed that particle numbers of small LDL and very small LDL were significantly reduced in the PABR group, whereas in the WR group, they were increased [113].

#### Chitosan

Chitosan is derived from chitin deacetylation and extracted from shellfish. It has anti-fungal, anti-bacterial, antioxidant activity and reduced fat absorption properties [114–116]. Several meta-analyses studies in patients with hypercholesterolemia [117] showed that chitosan only reduced total cholesterol In a pilot study, 28 patients with hypertriglyceridemia received 2 tablets daily (125 mg/d) of chitosan derived from fungal mycelium for 4 months. The results showed that LDL peak particle size and LDL subclasses did not differ between groups though the LDL-2 subclass was significantly increased after 4 months supplementation [118].

#### L-citrulline

L-citrulline is non-essential amino acid that found in abundance in watermelon. Potential beneficial effects on cardio-metabolic health [119, 120] have been reported. In a recent study, 22 patients with flow mediated dilation of the brachial artery (< 5.5%) were recruited take 800 mg/day of L-citrulline for 8 weeks. The results showed that L-citrulline supplementation had no effect on sdLDL levels [121].

#### Armolipid plus®

Armolipid Plus**®** tablets contain 200 mg of RYR (3 mg of monacolin K with citrinine and aflatoxins free), 500 mg of berberine, 10 mg of policosanols, 0.2 mg of folic acid, 2.0 mg of coenzyme Q10 and 0.5 mg of astaxanthin. In a comparative study, 30 patients with familial combined hyperlipidemia undertook a run-in period of 4 weeks of a normocaloric diet (54% carbohydrate, 16% protein, 30% fat, 45–55 g/day fiber), following which they were randomized to Armolipid Plus**®** (one tablet per day) or placebo (microcrystalline cellulose, iron oxide brown 70, Compritol E ATO and magnesium stearate with the same shape and taste) groups for 8 weeks. The results showed that Armolipid Plus**®** significantly decreased the LDL score compared with baseline and with the placebo group. Whilst mean LDL size significantly increased in the Armolipid Plus**®** group from baseline, there was no difference compared to placebo [122]. In another placebo-controlled trial, 147 individuals with metabolic syndrome were randomized into two groups, the intervention group received one tablet daily of Armolipid Plus**®** whilst the control group received a placebo tablet (microcrystalline cellulose, iron oxide brown) for 24 weeks. The results showed that patients who received Armolipid Plus**®** had significantly larger sdLDL-C sizes in comparison to baseline, though the differences between groups were not significant [84] (Table 6).

**Table 6:** The effect of other specific food ingredients or nutraceuticals on plasma concentration of small dense low density lipoprotein (LDL), LDL particle number, and LDL particle size

Author, Year	Intervention	Dose per day	Treatment duration	Subjects	Method of assessment	Main outcomes	Final effects of specific diet ingredients or nutraceuticals on LDL (number, size and concentration)
Shimada et al. 2004 [111]	Oolong tea	1000 ml/day	4 weeks	22 patients with coronary artery disease	Gradient gel electrophoresis	A significant increase in plasma LDL particle size levels was observed in oolong tea group compared with baseline (25*.*02 ± 0*.*67 nm versus 25*.*31 ± 0*.*60 nm)	LDL particle size **↑**
Araki et al. 2017 [113]	PABR, WR	400 g	12 weeks	42 prediabete, overweight patients	High-performance liquid chromatography with gel permeation columns	Changes in particle numbers of small LDL and very small LDL in the PABR group were − 13.1 ± 61.7 nM and − 9.0 ± 26.8 nM, whereas in the WR group, they were 35.1 ± 60.8 nM and 16.4 ± 32.6 nM	PABR: small LDL **↓** PABR: very small LDL **↓** WR: small LDL **↑** and WR: very small LDL **↑**
Rizzo et al. 2013 [118]	Chitosan	125 mg/day	4 months	28 patients with hypertriglyceridemia	PAGE	LDL peak particle size and LDL subclasses a mild increased and decreased, respectively but not statistically significant change.	LDL particle size — Small dense LDL —
Morita et al. 2013 [121]	L-citrulline	800 mg/day	8 weeks	22 patients with flow mediated dilation (FMD) of the brachial artery (< 5.5%)	Quantitative technique for LDL subfractionation using the lipoprint LDL system	No significant effect was observed on sdLDL levels after supplementation with L-citrulline.	Small dense LDL —
Gentile et al. 2015 [122]	Armolipid Plus**®**	1 tablet/day	4 weeks	30 patients with familial combined hyperlipidemia	Lipoprint System (electrophoresis of lipid-stained serum)	Armolipid Plus**®** significantly decreased LDL score (− 28%) compared with baseline and with placebo groups. Mean LDL size significantly increased in Armolipid Plus**®** groups from baseline (+ 2.7 Å) but between groups were not significant.	Small dense LDL**↓** LDL particle size **↑**

*sdLDL* small dense Low-Density Lipoprotein, *LDL-C* Low-density lipoprotein cholesterol, *PAGE* Polyacrylamide gradient gel electrophoresis, *PABR* Partially-abraded brown rice, *WR* White rice, *mg* milligrams, *nm* nanometer, *Å* Angstrom

## Conclusion

**Fig. 2:**
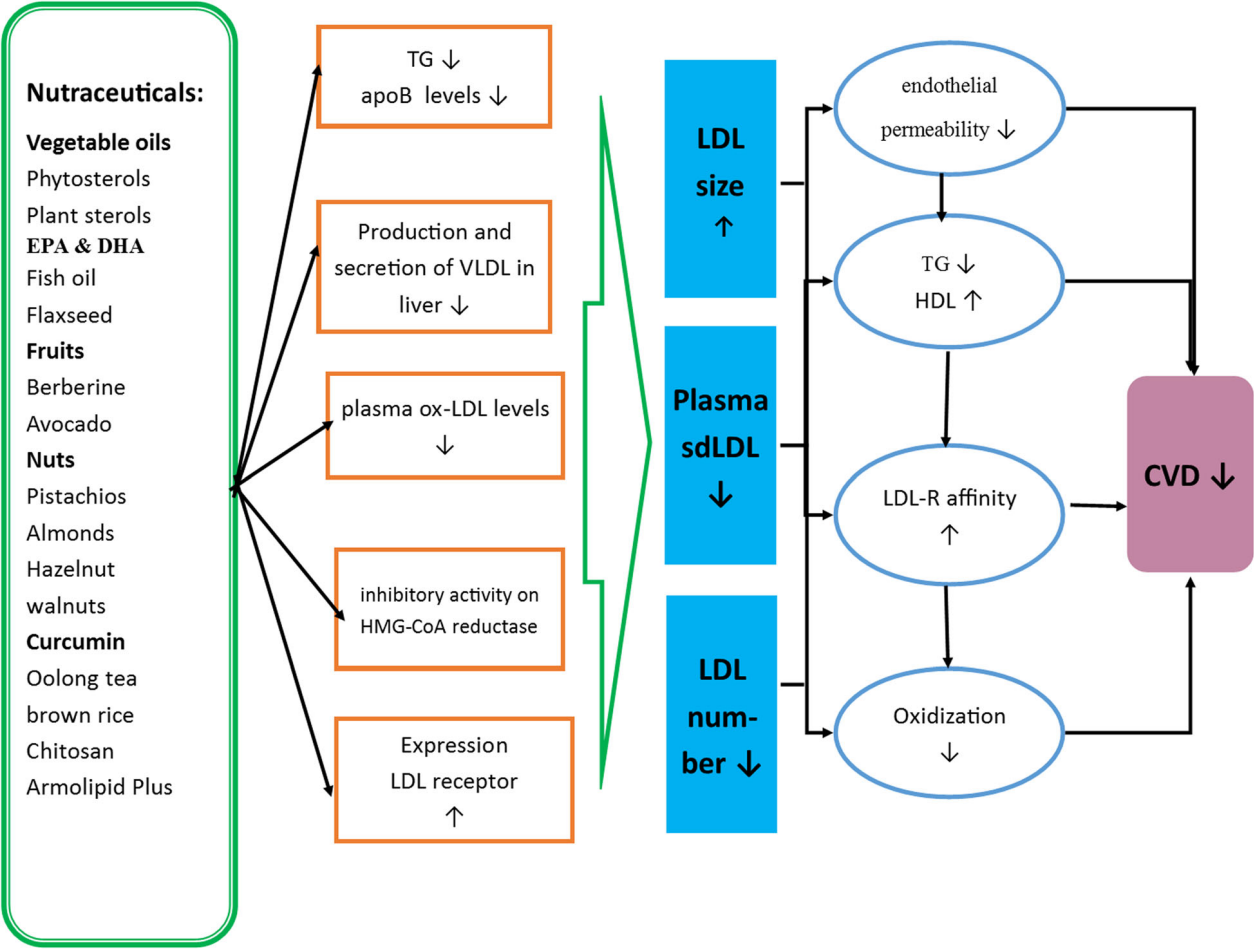
Schematic summary of pathways depicting the possible effects of nutraceuticals and specific diet ingredients on sdLDL reduction and its potential outcomes on cardiovascular diseases (CVD). sdLDL: small dense low density lipoprotein, Apo: apolipoprotein, VLDL: very-low-density lipoprotein, LDL-C: low-density lipoprotein cholesterol; CVD: cardiovascular diseases

This review has comprehensively assessed the effects of nutraceuticals and other diet ingredients on sdLDL, LDL particle number, and LDL particle size in human clinical trials. The results have shown that almost all of the nutraceuticals and specific diet ingredients discussed above such as omega-3 fatty acids have beneficial effects of LDL variants (Tables 1, 2, 3, 4, 5, and 6). Most of these agents including olive oil, phytosterols, psyllium, fish oil, EPA, DHA, flaxseed oil, berberine, avocado, strawberry, nuts (including pistachio, almonds and hazelnuts), curcumin and olong tea reduced sdLDL levels, LDL particle numbers or increased LDL particle size (Fig. 2). However, for many of the agents there are currently very few studies and the trials undertaken were often limited by study number, duration of the intervention or their design with few randomized clinical controlled trials. It has been reported that smaller and denser LDL particles have a higher susceptibility to oxidation and they are an independent atherogenic risk factor for CVD; therefore, larger clinical trials with plant-derived therapeutic agents and omega-3 fatty acids, or other specific ingredients of foods, are required to clarify and confirm their efficacy to lower the levels of sdLDL, LDL particle numbers or increase LDL particle size, and to determine optimal dosing strategies. However, this review does highlight that medicinal plants, nutraceuticals and omega-3 fatty acids may have a role as natural products that can be used as adjunct or complementary therapeutic agents to reduce sdLDL levels, LDL particle numbers or increase LDL particle size that may have a beneficial impact on CVD risk.

However, it should be noted that the included studies in this review did not assess whether improvement in sdLDL levels led to a clinical impact. In addition, measurement methods of sdLDL levels differed between studies and the results may not be directly comparable. Thus, it is recommended that future research focusing on the evaluation of the efficacy of food ingredients or nutraceuticals in populations with high sdLDL levels should be of sufficient power and of robust design to give definitive conclusions, and to determine sdLDL using standardized methodology.

## Data Availability

Not applicable.

## Abbreviations

CVDs: Cardiovascular diseases
LDL-C: low density lipoprotein cholesterol
sdLDL: small dense low-dense lipoprotein
HDL: High-density lipoproteins
lbLDL: Large buoyant
VLDL: Very low-density lipoproteins
CETP: Cholesteryl ester transfers protein
LPL: Lipoprotein lipase
HL: Hepatic lipase
IDL: Intermediate-density lipoprotein
IHD: Ischemic heart disease
CHD: Coronary heart disease
AACE: Association of Clinical Endocrinologists
MUFA: Monounsaturated fatty acids
PAGE: Polyacrylamide gradient gel electrophoresis
PABR: Partially-abraded brown rice
WR: White rice
EPA: Eicosapentaenoic acid
SB: Sucrose-sweetened beverage
LPCOO: Low-polyphenol-content olive oil
HPCOO: High-polyphenol-content olive oil
IEB: Nositol-enriched beverage
NMR: Nuclear magnetic resonance
FCHL: Familial combined hyperlipidemia
SBO: Soybean oil
RBO: Rice bran oil
MeDiet: Mediterranean diet
ALA: Alpha-linolenic acid
TG: Triglycerides
DHA: Docosahexaenoic acid
NAFLD: Non-alcoholic Fatty Liver Disease
VAP-II: Vertical Auto Profile II
ALM: Almonds
CHOC: Dark chocolate
EVOO: Extra-virgin olive oil
FMD: Flow mediated dilation
